# Effects of planting of two common crops, *Allium fistulosum* and *Brassica napus*, on soil properties and microbial communities of ginseng cultivation in northeast China

**DOI:** 10.1186/s12866-022-02592-0

**Published:** 2022-07-22

**Authors:** Xingbo Bian, Xiaohang Yang, Qiong Li, Xin Sun

**Affiliations:** 1 Jilin Medical University, Jilin, China; 2 Jilin Ginseng Academy, Changchun University of Chinese Medicine, Changchun, China

**Keywords:** Ginseng cultivation soil, Microbial community, *Allium fistulosum*, *Brassica napus*, Crop rotation

## Abstract

**Background:**

Long-term cultivation of ginseng can cause severe crop disorders and soil sickness. Crop rotation is an effective agricultural management measure to improve soil sustainability and decrease pathogens. However, the suitable ginseng rotation system and the changes in soil microbial community and soil characteristics under the rotation system need to be further explored.

**Methods:**

To explore suitable ginseng crop rotation systems and improve soil utilization, *Allium fistulosum* and *Brassica napus* were planted on ginseng cultivation soil for one year. The effects of the two crops on the chemical properties and enzyme activities of the ginseng cultivation soil were evaluated by chemical analysis. In addition, amplicon sequencing targeting 16 s rDNA genes of bacteria and ITS of fungi has been used to characterize the functional and compositional diversity of microbial communities.

**Results:**

The results elucidated that the levels of available phosphorus (AP) and available potassium (AK) in the soil increased significantly after one year of cultivation for both crops and *Allium fistulosum* cultivation may also have reduced soil salinity. In addition, the effects of the two crops on the activities of key soil enzymes were different. Catalase (CAT), urease (URE), and acid phosphatase (A-PHO) activities were significantly reduced and sucrase (SUC), and laccase (LAC) activities were significantly increased after *Allium fistulosum* planting. While A-PHO activity was significantly increased and LAC activity was significantly decreased after *Brassica napus* planting. *Allium fistulosum* significantly reduced the abundance of soil fungal communities. The cultivation of *Allium fistulosum* and *Brassica napus* significantly altered the composition of soil bacterial and fungal communities, where changes in the abundance of dominant microorganisms, such as *Ascomycota*, and *Mortierellomycota*, etc., were closely related to soil chemistry and enzyme activity. Moreover, both significantly reduced the abundance of the pathogenic fungus *Ilyonectria*.

**Conclusions:**

Our study clarified the effects of *Allium fistulosum* and *Brassica napus* on the microbial community and physicochemical properties of ginseng cultivated soil and provides a basis for the sustainable application of ginseng cultivation soil and the development of ginseng crop rotation systems.

**Supplementary Information:**

The online version contains supplementary material available at 10.1186/s12866-022-02592-0.

## Introduction

Ginseng (*Panax ginseng* Meyer) is a perennial herb of the Acanthopanax family, known as “the king of all herbs” because of its high medicinal value [1]. It is mainly grown in northeast China, Korea, and Japan and has become one of the most popular herbal medicines globally [2, 3]. However, ginseng cultivation is difficult because ginseng is a shade-loving crop, and it takes a long period (usually 4–5 years) to produce the highly valued roots [4]. Furthermore, the long-term cultivation of ginseng has a significant impact on soil microbial community and soil physical and chemical properties, and ginseng is also adversely affected by allelopathy or self-toxicity in the growth process [5]. As a result, serious obstacles to continuous cropping appeared in ginseng cultivation, which limited the reseeding and reduced the yield, resulting in substantial economic losses [6]. To date, there is no feasible and effective way to deal with the continuous cropping obstacle of ginseng.

To ensure the sustainable and healthy development of the ginseng industry and avoid the waste of land resources, scholars have conducted much research on the microbial community and soil properties related to ginseng cultivation soil and have been exploring soil improvement methods and reasonable crop rotation systems after ginseng cultivation [7, 8]. Studies have shown that ginseng allelopathy is related to phenolic acids, saponins, and other substances secreted by ginseng roots, but it is not completely clear [9, 10]. Moreover, it is difficult for some crops to grow healthily in soil planted with ginseng due to the comprehensive changes (including decreased fertility, changes in microbial composition, and changes in physical and chemical properties) that occur after the planting of ginseng [11]. In China, forest destruction for ginseng cultivation has been banned due to land use requirements for agricultural production and forestry. It is of great significance to establish a ginseng rotation system and land resource utilization to find crops that can grow normally and have potential improvement effects on the soil after ginseng planting.

Scientific crop rotation can significantly enhance soil fertility, and the benefits generally vary with crop rotation species via root architectures and plant characteristics [12]. Crop rotation also significantly impacts soil organic matter and thus soil properties, especially over long periods of agricultural activity [13, 14]. Therefore, appropriate rotation can substantially improve soil quality to offer an effective solution for problems caused by soil sickness under long-term ginseng cultivation, maintaining land productivity while limiting negative side effects on the environment. In previous studies, scholars have tried to grow crops such as *Zea mays*, *Gastrodia elata*, *Panax quiquefolium*, and *Taxus cuspidata* in ginseng cultivation soil to alleviate the obstacles to continuous cropping, and field experiments of crop rotation combinations such as ginseng-*Asarum europaeum* and ginseng-*Chelidonium majus* have also been conducted [15–19]. The results showed that crop rotation in ginseng cultivation soils is beneficial to improving soil structure and restoring soil fertility, which laid the foundation for the study of continuous crop obstacles in ginseng cultivation soils. More importantly, ginseng crop disorders are mainly manifested by soil acidification and increased soil-borne diseases, so it is essential to investigate the effects of crop planting on the chemical properties and microbial communities (especially ginseng pathogens) of ginseng cultivation soils.

*Allium fistulosum* is an important *allium* genus plant, which is a delicious food plant and also has rich pharmacological activities [20]. Because of *Allium fistulosum* good adaptability to the environment, it is popular and widely grown in northeast China. *Brassica napus* is a widely edible vegetable in China. In addition, it is also one of the most important oil crops, an essential part of China's international trade, providing high quality edible vegetable oil for human beings [21, 22]. In northeast China, these two crops are often grown on the soil where ginseng has been cultivated. However, the effects of the two on the soil fertility, physical and chemical properties and microbial community after ginseng cultivation have not been reported. Therefore, it is necessary to investigate whether *Allium fistulosum* or *Brassica napus* can be used in crop rotation to improve the soil for ginseng cultivation.

In this study, the ginseng cultivation soil in the producing areas of ginseng genuine medicinal materials (Fusong County, Jilin Province, China) was selected as the research object, and the hypothesis that *Allium fistulosum* and *Brassica napus* rotation improved the ginseng cultivation soil was examined. The specific objectives were to (1) investigate the effects of *Allium fistulosum* and *Brassica napus* on ginseng cultivation soil physicochemical properties; (2) analysis the effects of *Allium fistulosum* and *Brassica napus* on ginseng cultivation soil microbial composition and function, and (3) evaluate the comprehensive influence of *Allium fistulosum* and *Brassica napus* on soil quality of ginseng cultivation. This study elucidated the effects of *Allium fistulosum* and *Brassica napus* on microbial communities and physicochemical properties in ginseng cultivated soils and provided a basis for the improvement of ginseng cultivated soils and the establishment of suitable ginseng crop rotation systems.

## Results

### Soil properties and nutrients

Soil properties were determined one year after *Allium fistulosum* and *Brassica napus* cultivation (Table 1). The pH values of both *Allium fistulosum* (AF) and *Brassica napus* (BN) groups were not significantly different compared to the control check (CK) group. The electrical conductivity (EC) values in the AF group were substantially lower than those in the CK group; in contrast, the EC values in the BN group were significantly higher than those in the CK group. In terms of soil nutrients, there were no significant differences in organic matter (OM) and available nitrogen (AN) levels among the three groups of soil samples (Table 1). The effects of cultivation of the two plants on the AP and AK of ginseng cultivation soil were different, and the AP and AK levels of soil samples in the AF group were significantly higher than those in the CK and BN groups, and the AP in the BN group was also lower than that in the CK group (Table 1). The above results suggest that the cultivation of different plants can have different effects on the soil properties and fast-acting nutrients of ginseng cultivation soil.

**Table 1:** Physicochemical properties, nutrient content and enzyme activities of the soil in different treatments

		**CK**	**AF**	**BN**
**Soil properties and nutrients**	pH	5.508 ± 0.04**a**	5.475 ± 0.01**a**	5.483 ± 0.005**a**
	EC	44.575 ± 0.900**b**	42.125 ± 1.176**c**	51 ± 0.455**a**
	OM	41.788 ± 0.992**a**	41.960 ± 3.901**a**	38.607 ± 7.249**a**
	AN	313.25 ± 22.837**a**	323.575 ± 13.494**a**	308 ± 30.086**a**
	AP	11.346 ± 0.119**b**	12.141 ± 0.339**a**	8.997 ± 0.252**c**
	AK	184.205 ± 12.034**b**	233.46 ± 28.811**a**	216.555 ± 34.568**ab**
**Soil enzyme activity**	CAT	41.916 ± 1.062**a**	38.844 ± 2.307**b**	41.528 ± 1.027**a**
	URE	5519.757 ± 179.796**b**	5034.589 ± 52.889**c**	5889.081 ± 253.331**a**
	A-PHO	67,714.646 ± 1154.351**a**	61,855.498 ± 1176.980**b**	56,873.418 ± 1016.610**c**
	SUC	22.369 ± 1.545**b**	31.080 ± 2.518**a**	30.991 ± 2.297**a**
	LAC	24.049 ± 0.899**c**	30.480 ± 1.025**a**	27.106 ± 1.211**b**

*EC* Electric conductivity, *OM* Organic matter, *AN* available nitrogen, *AP* available phosphorus, *AK* Available potassium, *CAT* catalase, *URE* urease, *A-PHO* Acid phosphatase, *SUC* Sucrose, *LAC* Laccase. Error represents the standard deviation; different letters indicate a significant difference at the *P* < 0.05 level. Error represents the standard deviation; different letters indicate a significant difference at the *P* < 0.05 level

### Soil enzyme activities

We measured the activities of five critical enzymes in the soil after one year of *Allium fistulosum* and *Brassica napus* cultivation (Table 1). We found that the soil catalase (CAT) and urease (URE) activities in the AF group were significantly lower than those in the CK and BN groups. Meanwhile, there was no significant difference in CAT activity between the CK and BN groups, while the activity of URE in the BN group was significantly higher than in the CK group. The acid phosphatase (A-PHO) activity was significantly lower in the AF and BN groups than in the CK group, and it was also lower in the BN group than in the AF group. In contrast, the sucrase (SUC) and laccase (LAC) activities were significantly higher in the AF and BN groups than in the CK group, with the LAC in the AF group being even higher than that in the BN group.

### Microbial diversity and richness

To assess the effect of *Allium fistulosum* and *Brassica napus* cultivation on the diversity and richness of microorganisms in ginseng cultivation soil, we calculated alpha diversity indices for each group of soil samples. We compared five alpha diversity indices (Coverage, Chao, ACE, Shannon, and Simpson index) for each group of soil bacterial communities. Coverage is an estimate of sampling completeness, the Chao and Ace indices reflect microbial community richness, and the Simpson and Shannon indices are commonly used to assess community diversity [23–26]. Interestingly, there were no significant differences between the individual index for all samples (Table 2). This result indicates that the cultivation of *Allium fistulosum* and *Brassica napus* did not have a significant effect on the diversity and abundance of bacterial communities in ginseng cultivation soil.

**Table 2:** Soil bacterial and fungal alpha diversity in different treatments. Error represents the standard deviation; different letters indicate a significant difference at the P < 0.05 level

		CK	AF	BN
**bacteria**	shannon	6.19 ± 0.09**a**	6.20 ± 0.06**a**	6.25 ± 0.41**a**
	simpson	0.0069 ± 0.0005**a**	0.00709 ± 0.0007**a**	0.0072 ± 0.0039**a**
	ace	4593.4 ± 487.91**a**	4598.8 ± 162.45**a**	4960.2 ± 775.56**a**
	chao	4566.7 ± 476.88**a**	4581.8 ± 158.13**a**	4870.8 ± 759.62**a**
	coverage	0.9942 ± 0.0010**a**	0.9943 ± 0.0006**a**	0.9938 ± 0.0012**a**
**fungi**	shannon	4.82 ± 0.26**a**	4.57 ± 0.18**a**	3.35 ± 1.80**a**
	simpson	0.0275 ± 0.0119**a**	0.0345 ± 0.0133**a**	0.2167 ± 0.3406**a**
	ace	1741.6 ± 70.01**a**	1551.2 ± 64.48**b**	1207.7 ± 618.18**ab**
	chao	1723.8 ± 71.74**a**	1544.6 ± 53.64**b**	1199.7 ± 619.09**ab**
	coverage	0.9984 ± 0.0003**a**	0.9986 ± 0.0001**a**	0.9987 ± 0.0006**a**

In terms of fungi, there were no significant differences in Shannon, Simpson, and coverage indices between the groups. Whereas the ace and Chao indices of fungal communities were significantly lower in the AF group compared to the CK group, there was no difference in the BN group compared to it (Table 2). The above results then indicate that the cultivation of both plants had no significant effect on the diversity of soil fungal communities in ginseng cultivation, but the cultivation of *Allium fistulosum* may have significantly reduced the richness of soil fungal communities.

### Microbial community composition and structure

**Fig.1:**
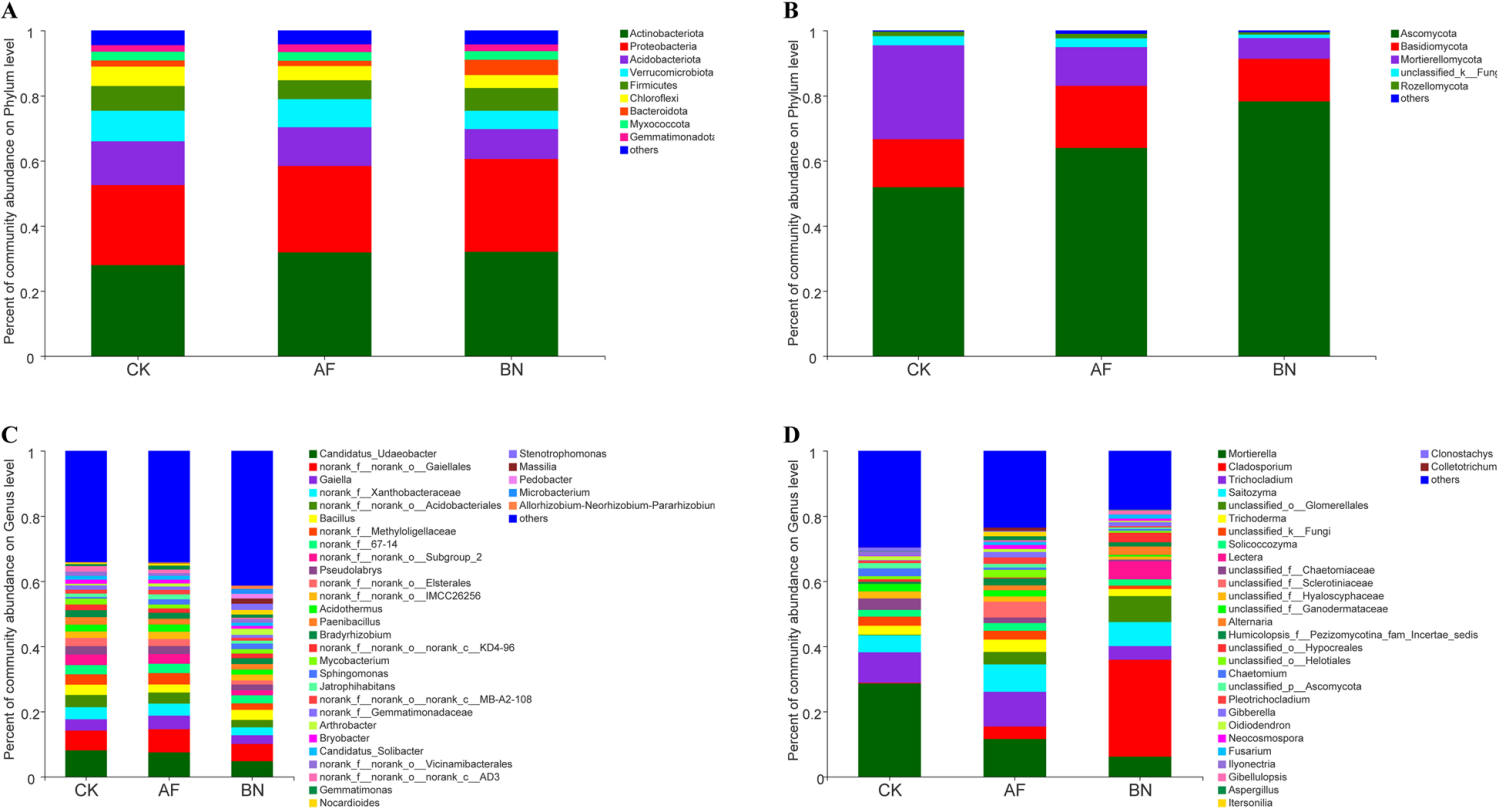
Relative abundances of (**A**) bacterial and (**B**) fungal phylum level and (**C**) bacterial and (**D**) fungal genus level in different treatments

To analyze the composition of soil microbial communities in each group, we counted the relative abundance of bacterial and fungal communities at the phylum level and genus level in each group. The relative abundance at the bacterial phylum level showed that *Actinobacteria* (30.60%) were the dominant phylum, followed by *Proteobacteria* (26.59%) and *Acidobacteriota* (11.50%) (Fig. 1A). The results of the analysis of the differences between bacterial groups at the level of each phylum are shown in Fig. S1, and no differences were found among the three groups for the main bacterial phylum. The most abundant bacterial at the genus level were *Candidatus Udaeobacter* (6.80%), followed by *norank-o-Gaiellales* (6.14%) and *Gaiella* (3.43%) (Fig. 1C).

In terms of fungi, the highest relative abundance at the phylum level was found in *Ascomycota* (64.70%), followed by *Basidiomycota* (15.68%) and *Mortierellomycota* (15.61%) (Fig. 1B). Significant analysis of the differences between groups is shown in Fig. S2, where we found significant differences in the relative abundance of *Ascomycota*, with the BN group having the highest relative abundance, followed by the AF and CK groups. At the genus level, the highest relative abundance was found for *Mortierella* (15.45%), followed by *Cladosporium* (11.20%) and *Trichocladium* (8.07%) (Fig. 1D).

**Fig. 2:**
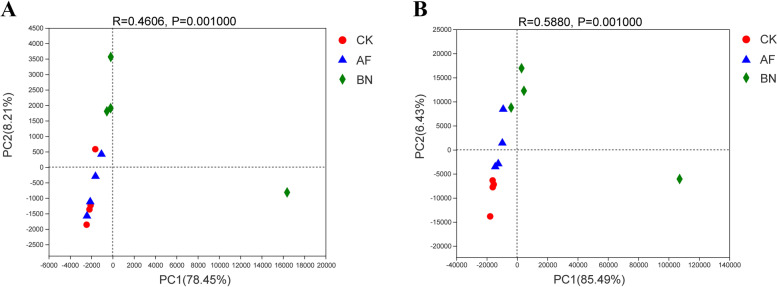
PCA of (**A**) bacterial and (**B**) fungal communities in different treatments

Principal components analysis (PCA) and ANOSIM at the OTU level clearly distinguished between soil bacterial (*R* = 0.4606, *P* = 0.001) and fungal (*R* = 0.5880, *P* = 0.001) communities in different groups (Fig. 2). The first two axes (PC1 and PC2) explained 78.45% and 8.21% in the bacterial community, respectively (Fig. 2A). The PCA of the fungal community was variance explained by PC1 and PC2 for 85.49% and 6.43%, respectively (Fig. 2B).

### Abundance of ginseng pathogens

**Fig. 3:**
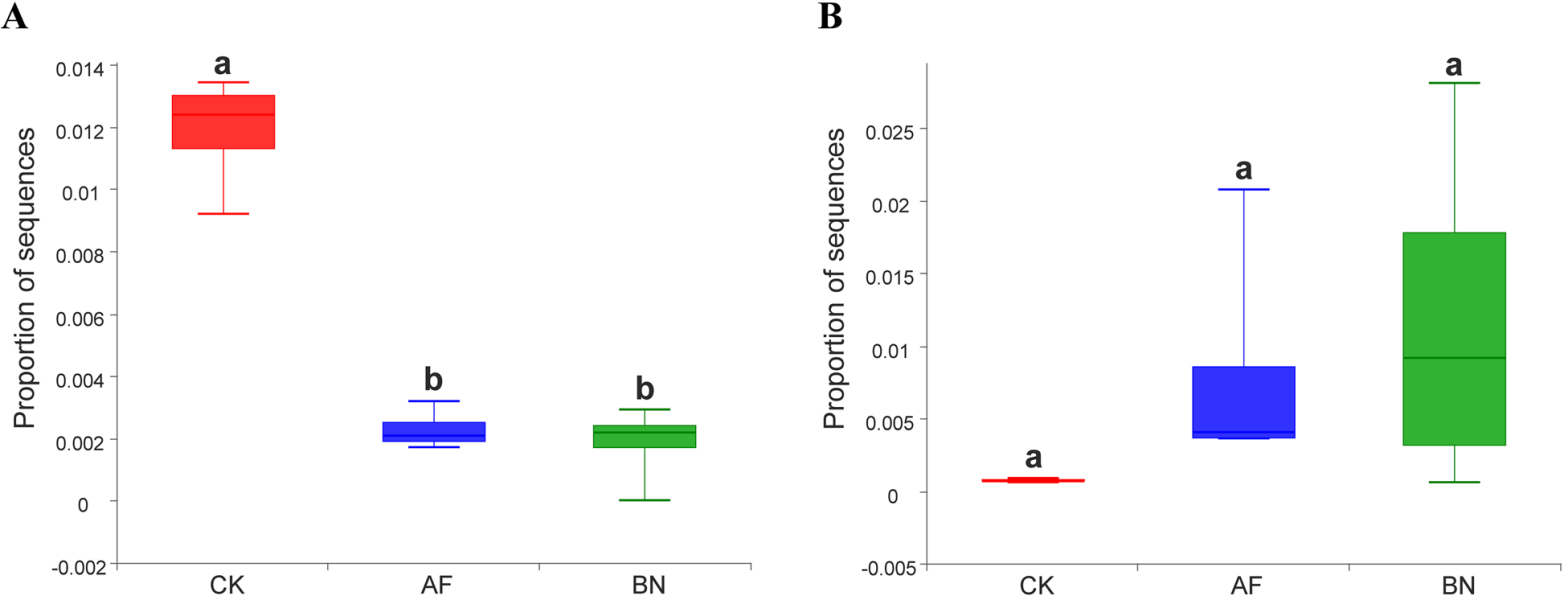
Relative abundances of (**A**) *Ilyonectria* and (**B**) *Fusarium*. Error represents the standard deviation; different letters indicate a significant difference at the *P* < 0.05 level

Here, we analyzed the abundance of these two major ginseng pathogenic fungi of the genera *Ilyonectria* and *Fusarium* in each group of soil samples. As shown in Fig. 3A, the cultivation of *Allium fistulosum* and *Brassica napus* significantly reduced the abundance of *Ilyonectria* in ginseng cultivation soil. In contrast, the *Fusarium* abundance in the AF and BN groups was not significantly different compared to the CK group (Fig. 3B).

### Biomarker microbes

**Fig. 4:**
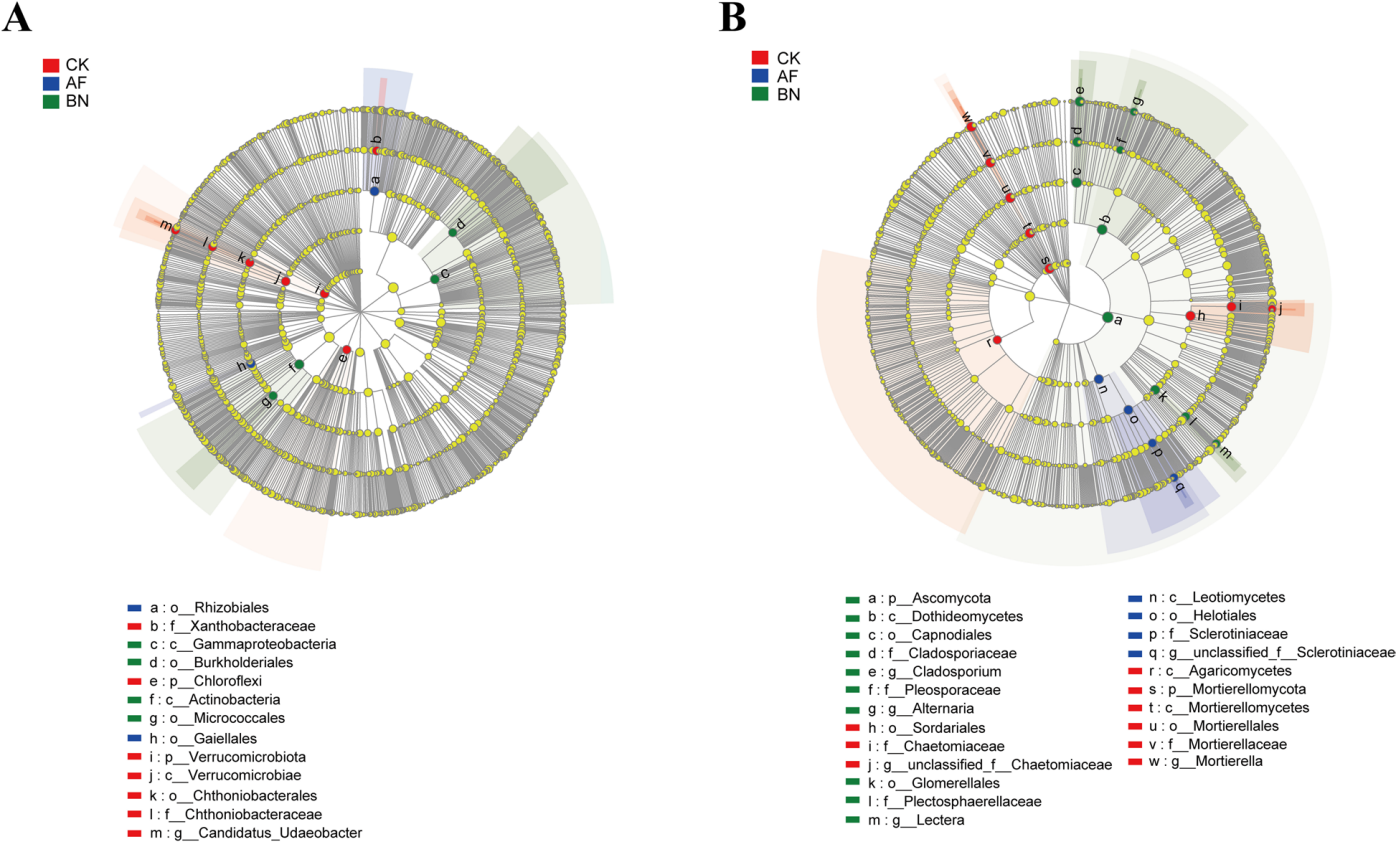
LEfSe analysis of (**A**) bacterial and (**B**) fungal communities in different treatments

Biomarker microbes were identified by LEfSe analysis for the microbial lineages from phylum to genus in different groups (LDA > 4). A total of 7 significant difference taxa were obtained in the CK group, including the phylum *Chloroflexi*, the phylum *Verrucomicrobiota* and the family *Xanthobacteraceae* (Fig. 4A). The significant different bacterial taxa of the AF group are order *Rhizobiales* and order *Gaiellales* (Fig. 4A). Furthermore, the significant difference bacterial taxa of the BN group are the class *Gammaproteobacteria* (including the order *Burkholderiales*) and the class *Actinobacteria* (including the order *Micrococcales*) (Fig. 4A).

For fungi, the significantly different taxa in the CK group were the phylum *Mortierellomycota*, the class *Agaricomycetes*, and the order *Sordariales* (Fig. 4B). The significantly different fungi taxa of the AF group are the class *Leotiomycetes* (including the order *Helotiales* and the family *Sclerotiniaceae*) (Fig. 4B). The significantly different fungi taxa of the BN group are the phylum *Ascomycota* (including the class *Dothideomycetes* and the order *Glomerellales*) (Fig. 4B). The LDA results for each group of bacterial and fungal communities are shown in Fig. S3 and Fig. S4, respectively.

### Relationship between Microbial community structure and soil properties

**Fig. 5:**
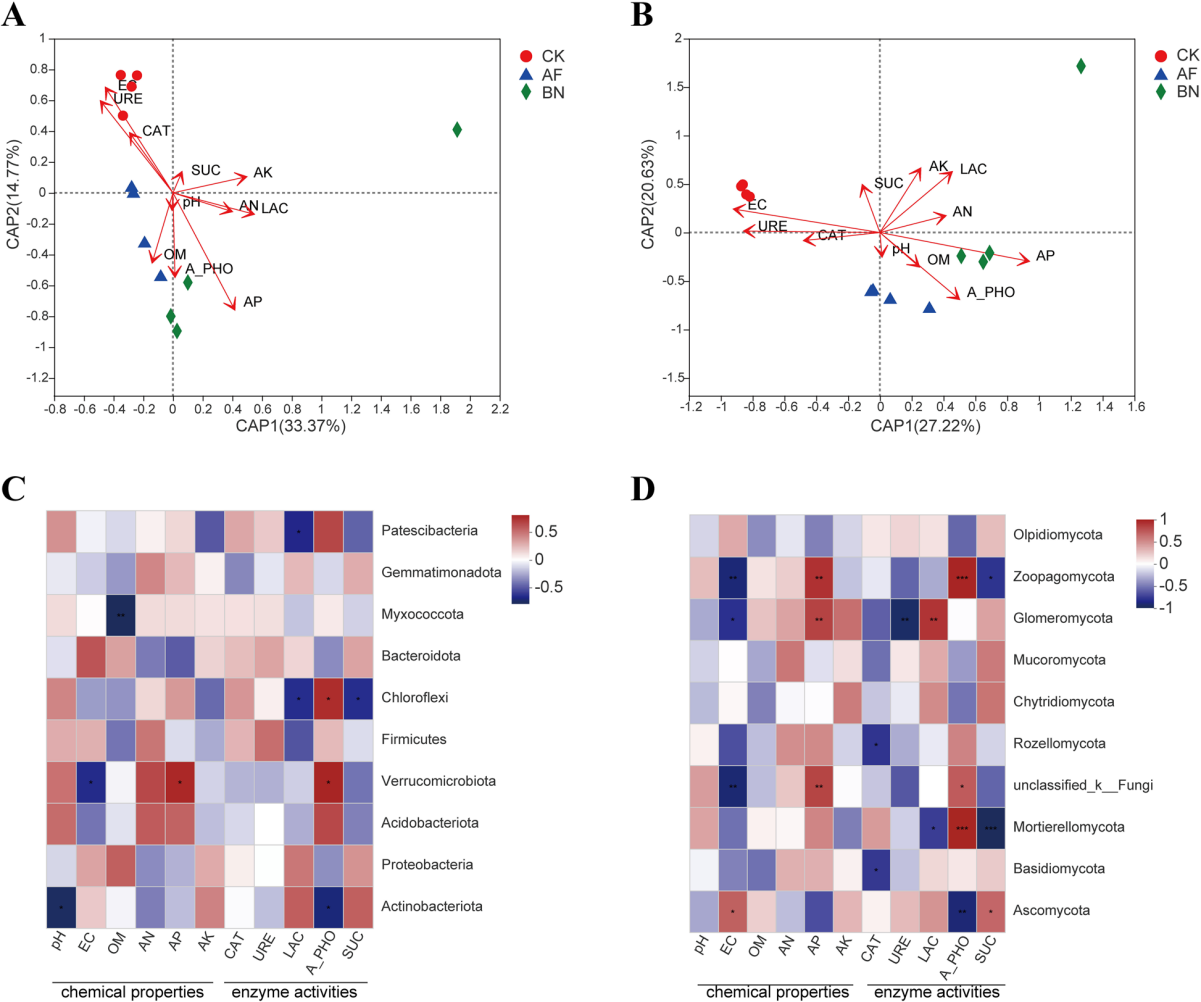
db-RDA analysis of (**A**) bacterial and (**B**) fungal and Pearson's correlation analysis of (**C**) bacterial and (**D**) fungal bacterial with soil variables. “*”, “**”, and “***” indicate a significant difference at the *P* < 0.05, *P* < 0.01 and *P* < 0.001 level, respectively.

Distance-based redundancy analysis (db-RDA) was used to analyze the binding contributions of soil chemical properties and soil enzyme activities to bacterial and fungal communities. The first two axes of the db-RDA model accounted for 48.14% and 47.85% of the total variance in the bacterial and fungal communities, respectively (Fig. 5A, B). Among these factors, EC and AP were the key chemical factors influencing the soil bacteria community. The degree of influence of chemical factors on the bacterial community was as follows: AP (R2 = 0.8994, *P* = 0.001) > EC (R2 = 0.8082, *P* = 0.003). In addition, the production rate and activity of soil enzymes are affected by microorganisms. In the db-RDA analysis results, URE activity may be significantly correlated with the bacterial community (R2 = 0.5076, *P* = 0.005) (Table S1). On the other hand, the db-RDA result showed that EC, AP, and AK were the dominant chemical factors influencing the fungal community. The degree of their influence is as follows: AP (R^2^ = 0. 0.9689, *P* = 0.001) > EC (R^2^ = 0.9033, *P* = 0.001) > AK (R^2^ = 0.4822, *P* = 0.048). In addition, the activity of three enzymes, including URE (R^2^ = 0. 7274, *P* = 0.003), A-PHO (R^2^ = 0. 7092, *P* = 0.005) and LAC (R^2^ = 0.5819, *P* = 0.016), may be significantly affected by the fungal community (Table S2).

The correlations between soil bacteria community at phylum levels and soil physicochemical characteristics and enzyme activities are shown in Fig. 5C. The abundance of bacteria phylum *Actinobacteriota* was significantly negatively correlated with the pH (*P* < 0.05) and A-PHO (*P* < 0.05). The abundance of *Verrucomicrobiota* was significantly negatively correlated with the EC (*P* < 0.05) and significantly positively correlated with the AP (*P* < 0.05) and A-PHO (*P* < 0.05). In terms of fungi, the abundance of *Ascomycota* was positively correlated with EC (*P* < 0.05) and SUC (*P* < 0.05) and negatively correlated with A-PHO (*P* < 0.01). *Basidiomycota* was negatively correlated with CAT (*P* < 0.05). *Mortierellomycota* was negatively correlated with LAC (*P* < 0.05) and SUC (*P* < 0.001), and positively correlated with A-PHO (*P* < 0.001) (Fig. 5D).

### Predictive analysis of Microbial community function

**Fig. 6:**
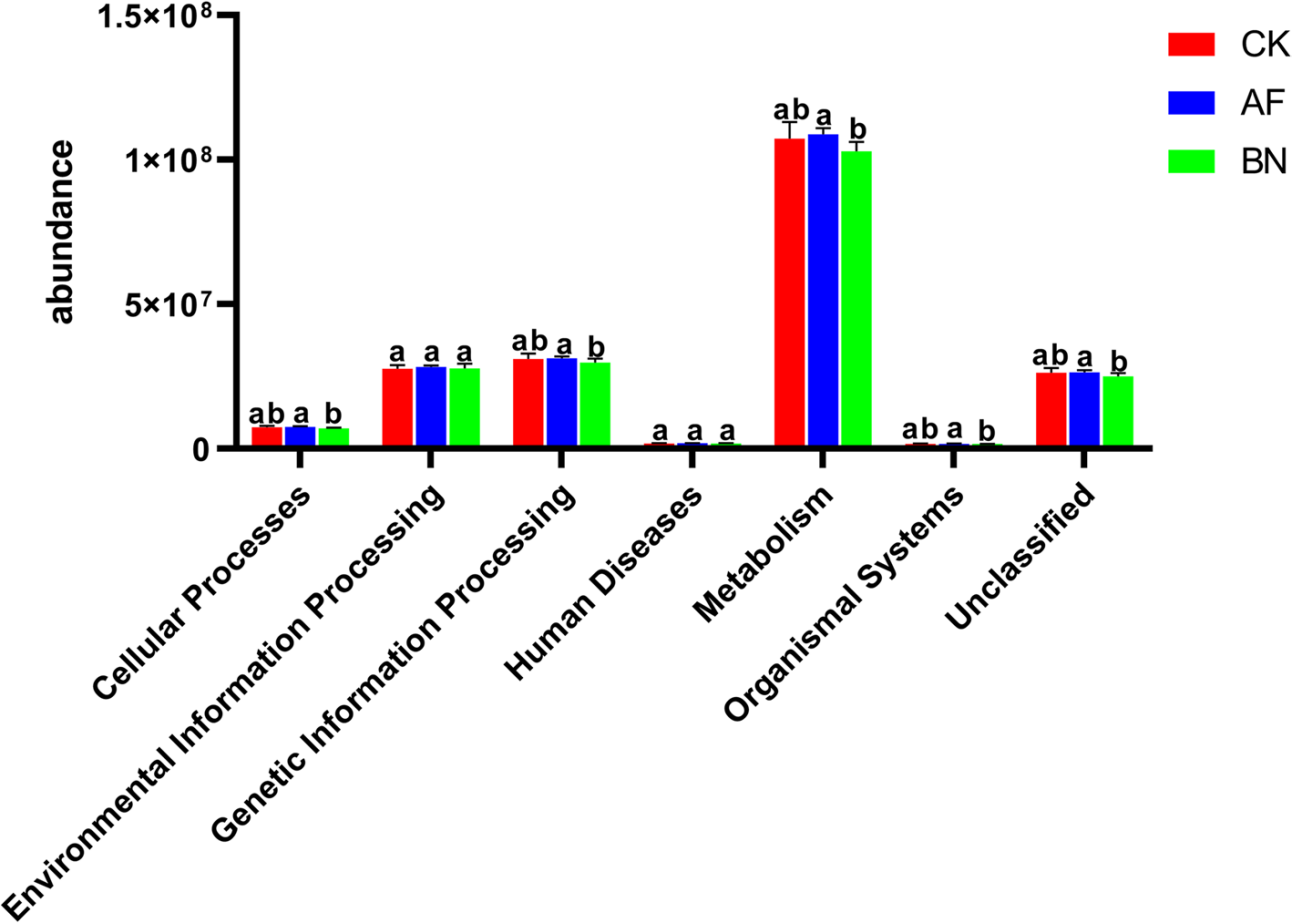
Bacterial function prediction of ginseng cultivation soil in different groups (hierarchy level 1). different letters indicate a significant difference at the *P* < 0.05 level

The OTUs abundance was normalized by PICRUSt, and the OTUs were annotated with KEGG functions to obtain the annotation information of OTUs at each KEGG function level and the abundance information of each function in different groups [27]. There are six primary functional layers (Cellular Processes, Environmental Information Processing, Genetic Information Processing, Human Diseases, Metabolism, and Organismal Systems) (Fig. 6). Also, there are 41 secondary functional layers (Table S3). Four of the six primary functional layers were significantly higher in AF than in the BN group, including Cellular Processes, Genetic Information Processing, Metabolism, and Organismal Systems (Fig. 6).

**Fig. 7:**
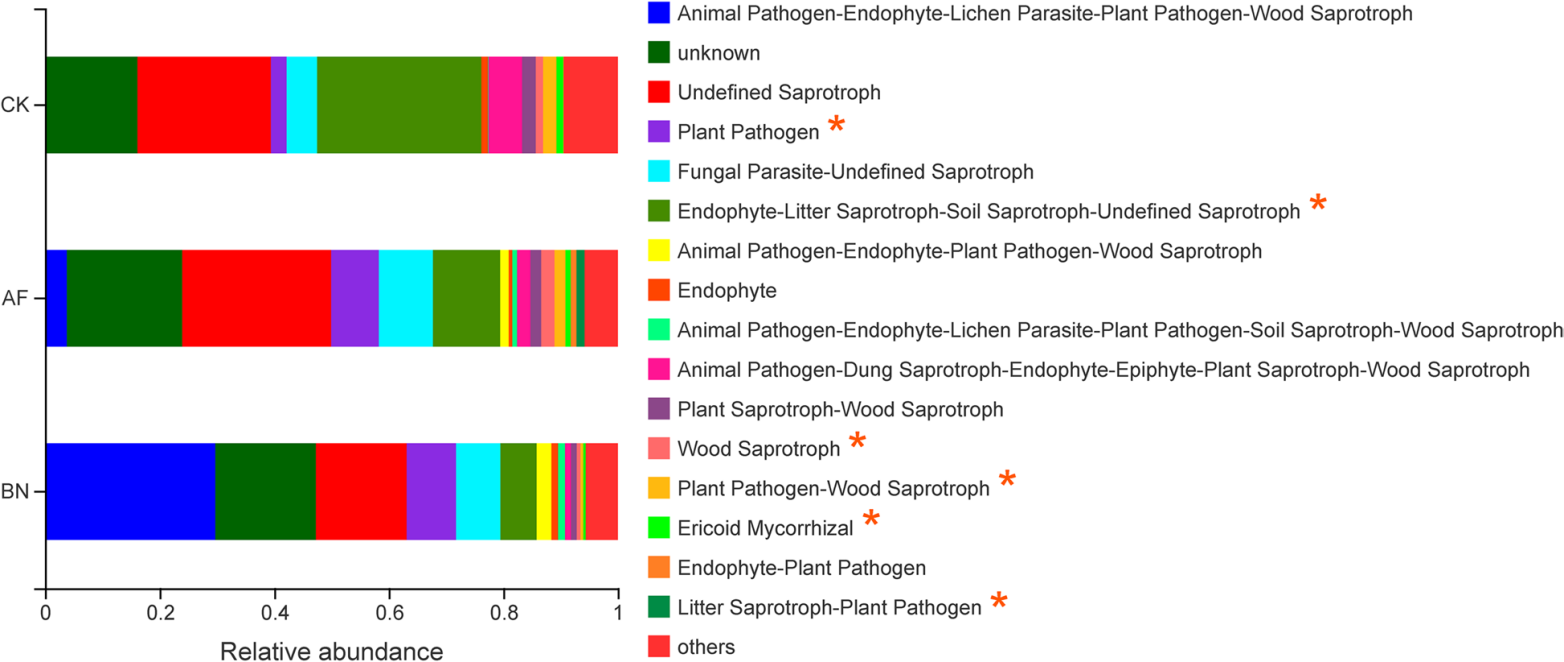
Normalized relative abundance of assigned fungal functional guilds. “*” indicate a significant difference at the *P* < 0.05 level

We used FunGuild to classify fungi, classifying the fungus OTUs into specific trophic groups and further subdividing it into specific ecological guilds [28, 29]. The guilds identified in this study are listed in Fig. 7. The cultivation of *Allium fistulosum* and *Brassica napus* changed the fungal function of ginseng cultivation soil. The fungal functional guilds with significant differences among each group are shown in Fig. S5, with substantial differences in the six guilds of Plant Pathogen, Endophyte-Litter Saprotroph-Soil Saprotroph-Undefined Saprotroph, Wood Saprotroph, Plant Pathogen-Wood Saprotroph, Ericoid Mycorrhizal, and Litter Saprotroph-Plant Pathogen after one year of cultivation of the two crops.

## Discussion

Multi-year cultivation of ginseng can lead to severe soil sickness, which in turn affects the sustainable application of the soil. Crop rotation is an effective agricultural management measure to alleviate soil sickness and improve soil application. Against the background that deforestation for ginseng cultivation is prohibited and the soil suitable for ginseng cultivation is limited, finding suitable crop rotation strategies has become an essential task for the sustainable development of the ginseng industry and improving soil utilization. In this study, we conducted one-year cultivation of *Allium fistulosum* and *Brassica napus* on ginseng cultivation soil in northeast China to analyze the effects of these two crops on the soil microbial and physicochemical properties.

*Allium fistulosum* and *Brassica napus* Affected the physicochemical characteristics and enzymatic activities of ginseng cultivation soil.

A variety of environmental factors are important indicators for evaluating soil quality [30]. A suitable pH is conducive to the growth of soil microorganisms [31]. Long-term ginseng cultivation lowers soil pH, leading to soil acidification. In addition, soil salinization is also a significant feature of soil degradation [32]. In our results, the one-year cultivation by *Allium fistulosum* and *Brassica napus* did not significantly change the pH of the ginseng cultivation soil. Usually, the higher the EC value, the more salinity there is in the soil, and the accumulation of salinity will lead to soil salinization [33]. However, we found that the cultivation of the two crops had different effects on soil EC (*Allium fistulosum* cultivation significantly decreased EC while *Brassica napus* significantly increased it). This means that *Allium fistulosum* reduces salinization in ginseng cultivation soil, while the opposite is true for *Brassica napus*. The possible reason is that the two crops (especially the roots) have different preferences for salt ions. Thus, leading to differences in the status and accumulation of salt ions (such as sodium) between the rhizosphere of different crops. Different plant species influence soil physicochemical properties through soil nutrient selectivity and/or chemosensory effects on soil microbial activity and abundance, thus affecting soil nutrient changes [34, 35]. Our study found that *Allium fistulosum* cultivation significantly increased the AP and AK of ginseng cultivation soil. Meanwhile, the AP of soils after *Brassica napus* cultivation was significantly lower than that of untreated ginseng cultivation soil. These results may be due to the different nutrient requirements of the two crops and the different effects of the two on soil microorganisms, which may also lead to differences in soil material cycling.

Several studies have shown that crop rotation can improve soil enzyme activity [36, 37]. In this study, *Allium fistulosum* cultivation reduced the activity of CAT, URE, and A-PHO in ginseng cultivation soil but increased the activity of SUC and LAC. The cultivation of *Brassica napus* had no significant effect on CAT activity but increased the activity of URE, SUC, and LAC and decreased the activity of A-PHO. Plants can alter nutrient cycling and energy metabolism by changing soil enzyme activity [38]. Moreover, soil enzyme activity is significantly and positively correlated with soil nutrient effectiveness [39]. Different soil enzymes have different effects on nutrient cycling [40]. CAT catalyzes the decomposition and transformation of peroxides, eliminating their adverse effects on soil health [41]. URE catalyzes the hydrolysis of urea and plays an essential role in soil N cycling [42]. The absorption and utilization of organic phosphorus in soil by crops require the action of PHO [43]. Soil SUC catalyzes the release of fructose and glucose from sucrose to provide a carbon source for plants and microorganisms, while LAC has an essential role in forming soil humus and organic matter [44, 45]. This result shows that the cultivation of *Allium fistulosum* and *Brassica napus* may positively affect the carbon cycle of ginseng cultivation soil. In addition, it is worth noting that, unlike most previous crop rotation studies, the effects of *Allium fistulosum* planting on soil enzymes of ginseng cultivation were not all positive. *Allium fistulosum* planting may have a potentially negative effect on soil nitrogen and phosphorus cycling and may also reduce the mitigation process of the toxic effects of hydrogen peroxide in the soil.

*Allium fistulosum* and *Brassica napus* affected the microbial communities of ginseng cultivation soil.

Plants regulate the activity of soil microorganisms through allelopathy and extension [46]. Therefore, different plant species usually recruit microbial groups. The diversity and richness of soil microorganisms after one year of *Allium fistulosum* and *Brassica napus* planting were analyzed by comparison of alpha diversity indices. Our results indicate that the cultivation of *Allium fistulosum* had no significant effect on the diversity and richness of soil bacterial communities but may have significantly reduced the richness of soil fungal communities. At the same time, one year of *Brassica napus* cultivation did not contribute to the diversity and richness of microorganisms in ginseng cultivation soil.

The bacterial phyla with high relative abundance in the three groups of soil samples were *Actinobacteriota*, *Proteobacteria*, and *Acidobacteriota*. These three bacterial phyla have critical ecological functions in the soil. It has been reported that the phylum *Actinobacteriota* contains a variety of OM-degrading bacteria that can maintain high levels of carbon sources in the soil, which is essential for the soil carbon cycle [47]. The *Proteobacteria* phylum has a strong metabolic diversity, and its members are essential for nutrient cycling in the soi [48]. Moreover, the *Acidobacteriota* phylum can degrade OM in the apoplast, which has a positive effect on maintaining an effective supply of nutrients to the soil [49]. In our study, the relative abundance of the three groups of major soil bacterial phyla did not differ significantly, suggesting that *Allium fistulosum* and *Brassica napus* cultivation may not have a significant effect on the composition of soil bacteria at the phylum level in ginseng cultivation.

At the fungal phylum level, there was a significant effect of *Allium fistulosum* and *Brassica napus* cultivation on the abundance of the major fungal phyla *Ascomycota* and *Mortierellomycota* in ginseng cultivation soil. In previous studies, *Ascomycota* and *Mortierellomycota,* as saprophytic fungi, played dominant roles in the decomposition of soil OM [50]. Interestingly, the changes in the abundance of *Ascomycota* and *Mortierellomycota* were reversed after one year of planting for both plants (*Ascomycota* abundance increased and *Mortierellomycota* abundance decreased). This may indicate that *Ascomycota* is more suitable for ginseng cultivation soil under both plant cultivation compared to *Mortierellomycota*.

In addition, attention was paid to the microbial composition of the three groups of soil samples at the genus level. We found that *Allium fistulosum* and *Brassica napus* plantings had significant effects on the genus level of soil microorganisms, no matter bacteria or fungi. Among them, we noted changes in the abundance of two important ginseng pathogenic fungal genera, *Ilyonectria* and *Fusarium*. The accumulation of pathogenic fungi in the soil is one of the main factors of ginseng continuous cropping obstacle and soil sickness. *Ilyonectria* and *Fusarium* are two important pathogenic fungi in the ginseng growth process, which can cause a variety of ginseng diseases [51, 52]. In our study, the abundance of *Ilyonectria* in ginseng cultivation soil was significantly reduced after one year of *Allium fistulosum* and *Brassica napus* cultivation, but the abundance of *Fusarium* was not significantly changed. *Ilyonectria* has been shown to produce specific metabolites, such as resorcylic acid lactones, to affect the plant immune system [53]. Therefore, the cultivation of both crops may reduce the accumulation of harmful plant substances in the ginseng cultivated soil, leading to increased soil health.

The composition of the bacterial and fungal communities was analyzed using the LEfSe tool to identify specific taxa in the different treatment groups [54]. In our results, both *Allium fistulosum* and *Brassica napus* produced their own significantly different taxa after one year of planting. These results further suggest that *Allium fistulosum* and *Brassica napu* cultivation may affect multiple taxa below the phylum level. The significant differences in soil microbial community composition of ginseng cultivation after different plant cultivation suggest the importance of plant rotation selection. The PCA and ANOSIM analysis results in this study provide ample evidence that *Allium fistulosum* and *Brassica napus* caused profound changes in the composition and structure of microbial communities in ginseng cultivation soil. In short, the cultivation of *Allium fistulosum* and *Brassica napus* for one year resulted in significant changes in the microbial community of the ginseng cultivation soil. Cultivation of *Allium fistulosum* and *Brassica napus* may alter soil material cycling and energy flow by changing the abundance of certain microbial taxa. The two crops do not have the same impact on the microbial community, which is related to their different allelopathy due to their different metabolic characteristics. Further, although the ecological functions of some of the microbial taxa that changed significantly after the cultivation of both crops are not known, the soil microbial network previously formed as a result of ginseng cultivation may have been altered, which could be beneficial for soil health as well as ecosystem sustainability [55].

### Correlations between microbial community structure and soil properties

There are close interactions between soils, microorganisms, and plants [56, 57]. The db-RDA results showed that the contribution of soil properties to the alteration of bacterial and fungal community composition was 48.14% and 47.85%, respectively. At the same time, it also revealed the dominant soil environmental factors in the construction of soil microbial community structure of ginseng cultivation. Pearson correlation analysis showed that soil variables such as pH, EC, AP, and multiple soil enzyme activities were significantly positively or negatively correlated with dominant bacterial and fungal phyla. *Actinobacteriota* showed a significant negative correlation with pH and A-PHO. *Verrucomicrobiota* showed a positive correlation with AP and A-PHO and a negative correlation with EC. In addition, *Chloroflexi* was significantly and positively correlated with A-PHO and negatively correlated with LAC and SUC. These results suggest that some critical chemical factors have important effects on bacteria communities in ginseng cultivation soil. Moreover, some soil enzymes, especially A-PHO, may be affected by the bacterial community. Chemical factors (EC, AP) and soil enzymes (CAT, LAC, A-PHO, and SUC) were significantly correlated with the fungal dominance phylum. In particular, *Mortierellomycota* and *Ascomycota* were strongly correlated with the activities of several soil key enzymes. In a word, different microorganisms are not equally sensitive to environmental factors. One year after *Allium fistulosum* and *Brassica napus* cultivation, some dominant bacteria and fungi in the soil were closely related to soil nutrients, chemical properties, and key enzyme activities. Further research is of great significance to the improvement of ginseng cultivation soil, the sustainability of ginseng production, and the maintenance of the soil environment.

## Conclusions

To explore the sustainable application of ginseng cultivation soil and an effective ginseng rotation system, we investigated the effects of *Allium fistulosum* and *Brassica napus* cultivation on the physicochemical properties and microbial communities of ginseng cultivation soil. The results showed that the chemical properties and soil microbial communities of ginseng cultivation soil changed significantly after one year of cultivation of both plants. *Allium fistulosum* cultivation increased soil AP and AK and decreased soil EC. While *Brassica napus* significantly reduced AP and also increased soil EC. Overall, *Allium fistulosum* improved soil nutrients and reduced salinization in ginseng cultivation soil, while *Allium fistulosum* cultivation increased SUC and LAC and reduced CAT, URE, and A-PHO. *Brassica napus* had no significant effect on soil bacterial and fungal community richness and diversity. Cultivation of *Allium fistulosum* and *Brassica napus* significantly altered soil microbial community composition, and these microbial alterations were associated with soil nutrient cycling. Moreover, both plants reduced the abundance of the ginseng pathogenic fungus *Ilyonectria*. Further studies should focus on soil enzyme activities as well as root exudate characteristics.

## Materials and methods

### Experimental site

The study field was located in Fusong County, Jilin Province, China (42.40′ N and 127.09′ E), and is one of the ginseng genuine medicinal production areas. The study field has a typical temperate monsoon climate with an altitude of 410 m. The average annual temperature and annual rainfall are 4 °C and 800 mm, respectively. Also, the site has an annual frost-free period of 120 days.

### Experimental design and sample collection

Ginseng was cultivated in the field for four years, from 2017 to 2020. Detailed information on fertilizer and pesticide application during ginseng cultivation was provided in the supplementary data. The pesticides were applied strictly according to the “ginseng safe production technical specification of pesticide application (DB22/T 1233–2019)”. After ginseng was collected in 2020, 12 plots of 10 square meters were divided, with 0.5 m intervals between adjacent plots.

There was a total of 3 treatments performed, each with 4 plots, including the CK group (ginseng cultivation soil without any treatment), AF group (cultivation of *Allium fistulosum*), and BN group (cultivation of *Brassica napus*). *Allium fistulosum* and *Brassica napus* seeds are purchased from Shouhe seeds Co. LTD (Shandong, China) and planted separately on the plots that had been divided. All crops were planted simultaneously and irrigated to ensure their survival during the beginning of the growing period. The crops were cultivated for one year, and no fertilization or other artificial intervention was used except weeding in all experimental plots.

After *Allium fistulosum* and *Brassica napus* had been grown for one year, soil samples were collected using a soil corer from each plot. All samples were collected on August 6, 2021. Here, five randomly collected soils (collected at a depth of 0–20 cm) in each plot were then mixed as one soil sample. A total of twelve soil samples were eventually obtained, four from each group. All soil samples are passed through a 2 mm sieve to remove stones, litter, and plant roots. Subsequently, each sample was then divided into two parts: one stored at 4 °C for analyzing the chemical and physical properties of the soil, and the other stored at -80 °C for microbial community analysis.

### Chemical analysis of the soil samples

The soil pH was measured at a soil: water ratio of 1:2.5 (w/v) using a PHS-3C pH meter (Leici, Shanghai, China). The soil EC was measured at a soil: water ratio of 1:5 (w/v) using a DDSJ-318 conductivity meter (Leici, Shanghai, China).

Soil OM was measured by the potassium dichromate oxidation-external heating method [58]. Soil AN was analyzed using the alkaline hydrolysis method [59]. Soil AP was determined by the NaHCO_3_ extraction molybdenum antimony colorimetry method [60]. Soil AK was determined by the NH_4_OAc extraction fame photometric method [61].

### Enzyme activity of soil samples

The activities of soil URE, A-PHO, SUC, CAT, and LAC were measured. The soil URE activity was determined by indophenol blue colorimetry; the A-PHO activity was determined by disodium phenyl phosphate colorimetry method; the SUC activity was determined by 3,5-dinitrosalicylic acid colorimetry; the activity of CAT was determined by KMnO_4_ titration; the LAC activity was determined by ABTS colorimetry [62, 63].

### DNA extracting and sequencing

In order to study the diversity and structure of soil bacterial and fungal communities, high-throughput sequencing of bacterial 16S ribosomal RNA (rRNA) gene and the Internal Transcribed Spacer (ITS) regions of the fungal gene was carried out on the Illumina MiSeq platform (Illumina, San Diego, USA). According to the manufacturer’s instruction, the bacterial and fungal DNA from 0.5 g of fresh soil samples was extracted using the E.Z.N.A. soil DNA Kit (Omega Bio-Tek, Norcross, GA, USA). The final DNA concentration and purification were evaluated by a NanoDrop 2000 UV − vis spectrophotometer (Thermo Scientific, Wilmington, USA), and DNA quality was checked by agarose gel electrophoresis.

The bacterial 16S rRNA genes and the fungal ITS rRNA genes were amplified respectively, and the primer information was as follows: 16S V3-V4 region primers were 338F (5′-ACTCCTACGGGAGGCAGCAG-3′) and 806R (5′-GGACTACHVGGGTWTCTAAT-3′); ITS region genes primers were ITS1F (5′-CTTGGTCATTTAGAGGAAGTAA-3′) and ITS2R (5′-GCTGCGTTCTTCATCGATGC-3′) [64, 65]. The PCR reaction system (20 μL) contained 5 × FastPfu buffer (4 μL), 2.5 mM dNTPs 2 μL, 5 μM primers (0.8 μL), FastPfu DNA polymerase (0.4 μL) and DNA template (1 μL, approximately 10–20 ng soil DNA). The PCR thermocycler program was conducted as follows: denaturation at 95 ℃ for 3 min, followed by 27 (16S V3-V4) or 35 (ITS) cycles at 95 ℃ for 30 s, 55 ℃ for 30 s, 72 ℃ for 45 s, and a final extension at 72 ℃ for 10 min. Then, the mixture of PCR products was extracted from a 2% agarose gel and further purified using the AxyPrep DNA Gel Extraction Kit (Axygen Biosciences, Union City, CA) [66]. Finally, sequencing was performed by Shanghai Majorbio Bio-pharm Biotechnology Co., Ltd. (Shanghai, China). The sequencing data have been uploaded to the National Center for Biotechnology Information (NCBI) database (Bacterial Accession Number: PRJNA821666; Fungal Accession Number: PRJNA821728).

### Bioinformatic analysis

Raw sequencing data were filtered to obtain high-quality reads with the QIIME (version 1.9.1, http://qiime.org/install/index) [67]. A total of 2.682.557 effective sequences of bacteria and 2.183.864 effective sequences of fungal were obtained from 12 soil samples of 3 groups. These sequences were clustered into different operational taxonomic units (OTUs) using UPARSE (version 7.1, http://www.drive5.com/uparse/) with > 97% sequence similarity [68]. The SILVA (http://www.arbsilva.de) and UNITE (http://unite.ut.ee/index.php) databases were selected as reference databases for analyses of bacterial and fungal taxonomies respectively, with a 70% confidence threshold [69].

To evaluate the coverage, richness, and diversity of soil microbial communities, we calculated the alpha diversity index, including Coverage, Chao1, ACE, Shannon, and Simpson, using Mothur (version 1.30.1) [70]. PCA at the OTU level was used to evaluate the differences in microbial communities between groups. Then, the indicator microbes of each group were found by linear discriminant analysis effect size (Lefse) analysis. The correlation between microbial community and soil variables was analyzed by db-RDA. Here, clustering analysis, PCA, Lefse, and db-RDA are performed on the free online Majorbio Cloud Platform (https://cloud.majorbio.com/).

PICRUSt predicted the soil bacterial function from 16S rRNA markers gene sequences, and the biological functions were annotated in the KEGG database [27]. In addition, the FUNGuild tool was used for the functional prediction and classification of soil fungi [29].

### Statistical analysis

Statistical analysis of the differences in soil properties and soil microbial alpha diversity was conducted by SPSS (version 19.0, Chicago, Illinois, USA). One-way ANOVA was used to identify significant differences among means, and a p < 0.05 was considered to indicate significance.

## Supplementary Information

**Additional file 1: Figure S1.** Analysis of differences in bacterial phylum levels. *: *P* < 0.05; **: *P* < 0.01.** Figure S2.** Analysis of differences in fungal phylum levels. *: *P* < 0.05; **: *P* < 0.01.** Figure S3.** The biomarker with LDA scores in bacterial communities associated with each treated soil from the three groups.** Figure S4.** The biomarker with LDA scores in fungi communities associated with each treated soil from the three groups.** Figure S5.** The fungal functional guilds with significant differences among the three groups.** Table S1. **Component extracted matrix of db-RDA for soil bacteria community.** Table S2. **Component extracted matrix of db-RDA for soil fungal community.** Table S3. **Bacterial function prediction of ginseng cultivation soil in different groups (hierarchy level 2).

## Data Availability

The sequencing data generated and analysed during the current study are available in the NCBI Sequence Read Archive database (https://www.ncbi.nlm.nih.gov/), Accession Number: PRJNA821666 and PRJNA821728.
