# Supplementary material for: Effects of planting of two common crops, Allium fistulosum and Brassica napus, on soil properties and microbial communities of ginseng cultivation in northeast China
Source: BMC Microbiol. 2022 Jul 22;22:182. doi: 10.1186/s12866-022-02592-0 (PMC9306067; doi:10.1186/s12866-022-02592-0)
Supplement: Supplementary file 1 — Additional file 1: Figure S1. Analysis of differences in bacterial phylum levels. *: P < 0.05; **: P < 0.01. Figure S2. Analysis of differences in fungal phylum levels. *: P < 0.05; **: P < 0.01. Figure S3. The biomarker with LDA scores in bacterial communities associated with each treated soil from the three groups. Figure S4. The biomarker with LDA scores in fungi communities associated with each treated soil from the three groups. Figure S5. The fungal functional guilds with significant differences among the three groups. Table S1. Component extracted matrix of db-RDA for soil bacteria community. Table S2. Component extracted matrix of db-RDA for soil fungal community. Table S3. Bacterial function prediction of ginseng cultivation soil in different groups (hierarchy level 2). [file 12866_2022_2592_MOESM1_ESM.zip › supplementary data .docx]

**Effects of planting of** **two common crops,** ***Allium fistulosum* and** ***Brassica napus*, on soil properties and microbial communities of ginseng cultivation in northeast China**

*Xingbo Bian ^1^, Xiaohang Yang ^1^, Qiong Li ^2^ and Xin Sun ^1, *^*

*1．Jilin Medical University, Jilin, China*

*2．Jilin Ginseng Academy, Changchun University of Chinese Medicine, Changchun, China*

***	Correspondence: sunxinbh@126.com (X.S.)

**Detailed information on fertilizer and pesticide application**：

During the ginseng cultivation period, bio-organic fertilizer was applied at a ratio of 1.5 kg per square meter and purchased from Jilin Shenhua Biotechnology Co., LTD (China). In addition, pesticides applied in ginseng cultivation include “carbendazim (systemic broad-spectrum fungicide)” purchased from Shandong Xinnongji Biotechnology Co. LTD (China), “tiandashenbao (plant cell membrane stationary agent)” purchased from Shandong Tianda Biopharmaceutical Co. LTD (China) and “metalaxyl (Phenylamide fungicides)” purchased from Alta Technologies Co. LTD (China).

**Figure S1.** Analysis of differences in bacterial phylum levels. *: P < 0.05; **: P < 0.01.

**Figure S2.** Analysis of differences in fungal phylum levels. *: P < 0.05; **: P < 0.01.

**Figure S3.** The biomarker with LDA scores in bacterial communities associated with each treated soil from the three groups.

**Figure S4.** The biomarker with LDA scores in fungi communities associated with each treated soil from the three groups.

**Figure S5.** The fungal functional guilds with significant differences among the three groups.

|  | CAP1 | CAP2 | r2 | p_values |
| --- | --- | --- | --- | --- |
| pH | -0.0582 | -0.9983 | 0.0101 | 0.943 |
| EC | -0.554 | 0.8325 | 0.8082 | 0.003 |
| OM | -0.2955 | -0.9553 | 0.2588 | 0.201 |
| AN | 0.9567 | -0.291 | 0.1966 | 0.399 |
| AP | 0.4813 | -0.8766 | 0.8994 | 0.001 |
| AK | 0.9785 | 0.2061 | 0.2962 | 0.163 |
| CAT | -0.5997 | 0.8002 | 0.2721 | 0.176 |
| URE | -0.6338 | 0.7735 | 0.709 | 0.005 |
| LAC | 0.9693 | -0.2458 | 0.3702 | 0.094 |
| A_PHO | 0.025 | -0.9997 | 0.3482 | 0.094 |
| SUC | 0.4044 | 0.9146 | 0.0202 | 0.944 |

**Table S1.** Component extracted matrix of db-RDA for soil bacteria community.

|  | CAP1 | CAP2 | r2 | p_values |
| --- | --- | --- | --- | --- |
| pH | 0.0535 | -0.9986 | 0.0487 | 0.783 |
| EC | -0.9671 | 0.2543 | 0.9033 | 0.001 |
| OM | 0.5737 | -0.819 | 0.1707 | 0.402 |
| AN | 0.9224 | 0.3863 | 0.1902 | 0.4 |
| AP | 0.9535 | -0.3013 | 0.9689 | 0.001 |
| AK | 0.3602 | 0.9329 | 0.4822 | 0.048 |
| CAT | -0.9853 | -0.1707 | 0.2249 | 0.267 |
| URE | -0.9998 | 0.0178 | 0.7274 | 0.003 |
| LAC | 0.5867 | 0.8098 | 0.5819 | 0.016 |
| A_PHO | 0.5869 | -0.8097 | 0.7092 | 0.005 |
| SUC | -0.2186 | 0.9758 | 0.2316 | 0.325 |

**Table S2.** Component extracted matrix of db-RDA for soil fungal community.

| **Pathway level 1** | **Pathway level 2** | **CK** | **AF** | **BN** |
| --- | --- | --- | --- | --- |
| Metabolism | Amino Acid Metabolism | 22467139 | 22860203 | 21839006 |
| - | Biosynthesis of Other Secondary Metabolites | 2357633 | 2346583 | 2209476 |
| - | Cancers | 257409 | 262035 | 260352 |
| Metabolism | Carbohydrate Metabolism | 22198018 | 22516389 | 21400934 |
| - | Cardiovascular Diseases | 49397 | 50208 | 41288 |
| - | Cell Communication | 640 | 614 | 482 |
| Cellular Processes | Cell Growth and Death | 1154226 | 1166630 | 1077569 |
| Cellular Processes | Cell Motility | 5446633 | 5603936 | 5225352 |
| - | Cellular Processes and Signaling | 6443430 | 6466005 | 6174309 |
| Organismal Systems | Circulatory System | 50567 | 53214 | 52015 |
| Organismal Systems | Digestive System | 47660 | 46749 | 58061 |
| Organismal Systems | Endocrine System | 949260 | 981290 | 919291 |
| Metabolism | Energy Metabolism | 11362606 | 11478749 | 10774768 |
| Organismal Systems | Environmental Adaptation | 273366 | 276525 | 267314 |
| - | Enzyme Families | 4025585 | 4017161 | 3811027 |
| Organismal Systems | Excretory System | 121908 | 122674 | 105449 |
| Genetic Information Processing | Folding, Sorting and Degradation | 4406666 | 4428796 | 4221933 |
| - | Genetic Information Processing | 4599283 | 4566139 | 4255980 |
| Metabolism | Glycan Biosynthesis and Metabolism | 4189695 | 4090277 | 3694948 |
| Organismal Systems | Immune System | 50673 | 47190 | 62044 |
| - | Immune System Diseases | 111836 | 115821 | 110158 |
| - | Infectious Diseases | 763021 | 762459 | 710477 |
| Metabolism | Lipid Metabolism | 8871292 | 8998509 | 8328324 |
| Environmental Information Processing | Membrane Transport | 22888668 | 23461946 | 23208964 |
| - | Metabolic Diseases | 166347 | 167692 | 161665 |
| - | Metabolism | 5104011 | 5112674 | 4879215 |
| - | Metabolism of Cofactors and Vitamins | 8230388 | 8353911 | 7953778 |
| - | Metabolism of Other Amino Acids | 4075598 | 4177429 | 3969068 |
| - | Metabolism of Terpenoids and Polyketides | 4912826 | 4995288 | 4690592 |
| Organismal Systems | Nervous System | 206162 | 211310 | 199985 |
| - | Neurodegenerative Diseases | 503379 | 517129 | 494964 |
| Metabolism | Nucleotide Metabolism | 6141235 | 6203502 | 5988430 |
| - | Poorly Characterized | 10211403 | 10318281 | 9766049 |
| Genetic Information Processing | Replication and Repair | 13306596 | 13419361 | 12933590 |
| Organismal Systems | Sensory System | 206 | 200 | 148 |
| Environmental Information Processing | Signal Transduction | 4358435 | 4427206 | 4159079 |
| Environmental Information Processing | Signaling Molecules and Interaction | 430022 | 426703 | 406130 |
| Genetic Information Processing | Transcription | 5365611 | 5384666 | 5029402 |
| Genetic Information Processing | Translation | 8025315 | 8059744 | 7662609 |
| Cellular Processes | Transport and Catabolism | 811720 | 822687 | 760523 |
| Metabolism | Xenobiotics Biodegradation and Metabolism | 8358851 | 8630636 | 8221545 |

**Table S3.** Bacterial function prediction of ginseng cultivation soil in different groups (hierarchy level 2).
